# Supplementary material for: Heat adaptation of phage T7 under an extended genetic code
Source: Virus Evol. 2021 Dec 1;7(2):veab100. doi: 10.1093/ve/veab100 (PMC8923235; doi:10.1093/ve/veab100)
Supplement: veab100_Supp [file veab100_supp.zip › Sequencing_Analysis.zip!/Sequencing_Analysis/S3/evidence/MC_SIDE_1_43.html]

BRESEQ :: Evidence


| Predicted mutation | | | | | | |
| --- | --- | --- | --- | --- | --- | --- |
| evidence | seq id | position | mutation | annotation | gene | description |
| MC JC | NC\_001604.1 | 13,579 | Δ769 bp | intergenic (–/–) | – / – | –/– |

| Missing coverage evidence... | | | | | | | | | | |
| --- | --- | --- | --- | --- | --- | --- | --- | --- | --- | --- |
|  |  |  | seq id | start | end | size | ←reads | reads→ | gene | description |
| \* | \* | ÷ | NC\_001604.1 | 13579 | 14347 | 769 | 1841 [22] | [5] 1821 | *–/–* | –/– |

| New junction evidence | | | | | | | | | | | |
| --- | --- | --- | --- | --- | --- | --- | --- | --- | --- | --- | --- |
|  | | seq id | position | reads (cov) | reads (cov) | score | skew | freq | annotation | gene | product |
| \* | ? | NC\_001604.1 | = 13578 | 22 (0.010) | 1735 (1.040) | 298/584 | 0.1 | 99.3% | intergenic (–/–) | *–/–* | –/– |
|  | ? | NC\_001604.1 | 14348 = | 4 (0.000) |  |  |  |  | intergenic (–/–) | *–/–* | –/– |

Only 100 of 1841 total aligned reads displayed.  

`TGACTTTCCAGACGACTACGAGAAGTTTCGCTGGAGAGTCCCATTCTAATACGACTCACTAAAGGAGACACACCATGTTCAAACTGATTAAGAAGTTAGGCCAACTGCTGGTTCGTATGTACAACGTGGAAGCCAAGCGACTGAACGATGAGGCTCGTAAAGAGGCCACACAGTCACGCGCTCTGGCGATTCGCTCCAACGAACTGGCTGACAGTGCATCCACTAAAGTTACCGAGGCTGCCCGTGTGGCAAACCAAGCTCAACAGCTTTCCAAATTCTTTGAGTAATCAAACAGGAGAAACCATTATGTCTAACGTAGCTGAAACTATCCGTCTATCCGATACAGCTGACCAGTGGAACCGTCGAGTCCACATCAACGTTCGCAACGGTAAGGCGACTATGGTTTACCGCTGGAAGGACTCTAAGTCCTCTAAGAATCACACTCAGCGTATGACGTTGACAGATGAGCAAGCACTGCGTCTGGTCAATGCGCTTACCAAAGCTGCCGTGACAGCAATTCATGAAGCTGGTCGCGTCAATGAAGCTATGGCTATCCTCGACAAGATTGATAACTAAGAGTGGTATCCTCAAGGTCG  >  NC_001604.1/13278‑13873`  

                                                                                                                                                                                                                                                                                                            `|`                                                                                                                                                                                                                                                                                                         
       `gagacagactacgagAAGTTTCGCTGGTGAGTCCCATTCTAATACGACTCACTAAAGGAGACACACCATGTTCAAACTGATTAAGAAGTTAGGCCAACTGCTGGTTCGTATGTGCAACGTGGAAGCCAAGCGACTGAACGATGAGGCTCGTAAAGAGGCCACACAGTCACGCGCTCTGGCGATTCGCTCCAACGAACTGGCTGACAGTGCATCCACTAAAGTTACCGAGGCTGCCCGTGTGGCAAACCAAGCTCAACAGCTTTCCAAATTCTTTGAGTAATCAAACAGGAGAAAtcaatat                                                                                                                                                                                                                                                                                                  <  1:176294‑M1/295‑8 (MQ=255)`  
       `ccAGACGACTACGAGAAGTTTCGCTGGAGAGTCCCATTCTAATACGACTCACTAAAGGAGACACACCATGTTCAAACTGATTAAGAAGTTAGGCCAACTGCTGGTTCGTATGTACAACGTGGAAGCCAAGCGACTGAACGATGAGGCTCGTAAAGAGGCCACACAGTCACGCGCTCTGGCGATTCACTCCAACGAACTGGCTGACAGTGCATCCACTAAAGTTACCGAGGCTGCCCGTGTGGCAAACCAAGCTCAACAGCTTTCCAAATTCTTTGAGTAATCAAACAGGAGAAAtcaatat                                                                                                                                                                                                                                                                                                  <  1:35310‑M1/301‑8 (MQ=255)`  
          `gacgccTACGAGAAGTTTCGCTGGAGGGTCCCATTCTAATACGACTCACTAAAGGAGACACACCATGTTCAAACTGATTAAGAAGTTAGGCCAACTGCTGGTTCGTATGTACAACGTGGAAGCCAAGCGACTGAACGATGAGGCTCGTAAAGAGGCCACACAGTCACGCGCTCTGGCGATTCGCTCCAACGAACTGGCTGACAGTGCATCCACTAAAGTTACCGAGGCTGCCCGTGTGGCAAACCAAGCTCAACAGCTTTCCAAATTCTTTGAGTAATCAAACAGGAGAAAtcaatatgat                                                                                                                                                                                                                                                                                               <  1:100087‑M1/301‑11 (MQ=255)`  
                    `agaAGTTTCGCTGGAGAGGCACATTCTAATACGAATCACTAAAGGAGACACACCATGTTCAAACTGATTAAGAAGTGAGGCAAACTGCTGGTTCGTATGTACAACGTGGAAGCCAAGCGACTGAACGATGAGGCTCGTAAAGAGGCCACACAGTCACGCGCTCTGGCGATTCGCTCCAACGAACTGGCTGACAGTGCATCCACTAAAGTTACCGAGGCTGCCCGTGTGGCAAACCAAGCTCAACAGCTTTCCAAATTCTTTGAGTAATCAAACAGGAGAAAccattatgatcgtttctgac                                                                                                                                                                                                                                                                                     <  1:81729‑M1/301‑21 (MQ=255)`  
                           `tCGCTGGAGAGTCCCATTCTAATACGACTCACTAAAGGAGACACACCATGTTCAAACTGATTAAGAAGTTAGGCCAACTGCTGGTTCGTATGTACAACGTGGAAGCCAAGCGACTGAACGATGAGGCTCGTAAAGAGGCCACACAGTCACGCGCTCTGGCGATTCGCTCCAACGAACTGGCTGACAGTGCATCCACTAAAGTTACCGAGGCTGCCCGTGTGGCAAACCAAGCTCAACAGCTTTCCAAATTCTTTGAGTAATCAAACAGGAGAAAtcaatatgatcgtttctgacatcgaag                                                                                                                                                                                                                                                                              >  1:242567‑M1/1‑274 (MQ=255)`  
                                `ggAGAGTCCCATTCTAATACGACTCACTAAAGGAGACACACCATGTTCAAACTGATTAAGAAGTTAGGCCAACTGCTGGTTCGTATGTACAACGTGGAAGCCAAGCGACTGAACGATGAGGCTCGTAAAGAGGCCACACAGTCACGCGCTCTGGCGATTCGCTCCAACGAACTGGCTGACAGTGCATCCACTAAAGTTACCGAGGCTGCCCGTGTGGCAAACCAAGCTCAACAGCTTTCCAAATTCTTTGAGTAATCAAACAGGAGAAAccattatgatcgtttctgacatcgaagctaac                                                                                                                                                                                                                                                                         >  1:6747‑M1/1‑269 (MQ=255)`  
                                      `tCCCATACTAATACGACTCACTAAAGGAGACACACCATGTTCAAACTGATTAAGAAGTTAGGCCAACTGCTGGTTCGTATGTACAACGTGGAAGCCAAGCGACTGAACGATGAGGCTCGTAAAGAGGCCACACAGTCACGCGCTCTGGCGATTCGCTCCAACGAACTGGCTGACAGTGCATCCACTAAAGTTACCGAGGCTGCCCGTGTGGCAAACCAAGCTCAACAGCTTTCCAAATTCTTTGAGTAATCAAACAGGAGAAAtcaatatgatcgtttctgacatcgaagctaacgccctc                                                                                                                                                                                                                                                                   <  1:190558‑M1/301‑39 (MQ=255)`  
                                          `aTTCTAATACGACTCACTAAAGGAGACACACCATGTTCAAACTGATTAAGAAGTTAGGCCAACTGCTGGTTCGTATGTACAACGTGGAAGCCAAGCGACTGAACGATGAGGCTCGTAAAGAGGCCACACAGTCACGCGCTCTGGCGATTCGCTCCAACGAACTGGCTGACAGTGCATCCACTAAAGTTACCGAGGCTGCCCGTGTGGCAAACCAAGCTCAACAGCTTTCCAAATTCTTTGAGTAATCAAACAGGAGAAAccattatgatcgtttctgacatcgaagctaacgccctcttag                                                                                                                                                                                                                                                               <  1:168121‑M1/301‑43 (MQ=255)`  
                                           `ttCTAATACGACTCACTAAAGGAGACACACCATGTTCAAACTGATTAAGAAGTTAGGCCAACTGCTGGTTCGTATGTACAACGTGGAAGCCAAGCGACTGAACGATGAGGCTCGTAAAGAGGCCACACAGTCACGTGCTCTGGCGATTCGCTCCAACGAACTGGCTGACAGTGCATCCACTAAAGTTACCGAGGCTGCCCGTGTGGCAAACCAAGCTCAACAGCTTTCCAAATTCTTTGAGTAATCAAACAGGAGAAAtcaatatgatcgtttctgacatcgaagctaacgccctcttaga                                                                                                                                                                                                                                                              >  1:309437‑M1/1‑258 (MQ=255)`  
                                           `ttCTAATACGACTCACTAAAGGAGACACACCATGTTCAAACTGATTAAGAAGTTAGGCCAACTGCTGGTTCGTATGTACAACGTGGAAGCCAAGCGACTGAACGATGAGGCTCGTAAAGAGGCCACACAGTCACGCGCTCTGGCGATTCGCTCCAACGAACTGGCTGACAGTGCATCCACTAAAGTTACCGAGGCTGCCCGTGTGGCAAACCAAGCTCAACAGCTTTCCAAATTCTTTGAGTAATCAAACAGGAGAAAtcaatatgatcgtttctgacatcgaagctaacgccctcttaga                                                                                                                                                                                                                                                              >  1:172739‑M1/1‑258 (MQ=255)`  
                                           `ttCTAATACGACTCACTAAAGGAGACACACCATGTTCAAACTGATTAAGAAGTTAGGCCAACTGCTGGTTCGTATGTACAACGTGGAAGCCAAGCGACTGAACGATGAGGCTCGTAAAGAGGCCACACAGTCACGCGCTCTGGCGATTCGCTCCAACGAACTGGCTGACAGTGCATCCACTAAAGTTACCGAGGCTGCCCGTGTGGCAAACCAAGCTCAACAGCTTTCCAAATTCTTTGAGTAATCAAACAGGAGAAAccattatgatcgtttctgacatcgaagctaacgccctcttagc                                                                                                                                                                                                                                                              >  1:139405‑M1/1‑258 (MQ=255)`  
                                           `ttCTAATACGACTCACTAAAGGAGACACACCATGTTCAAACTGATTAAGAAGTTAGGCCAACTGCTGGTTCGTATGTACAACGTGGAAGCCAAGCGACTGAACGATGAGGCTCGTAAAGAGGCCACACAGTCACGCGCTCTGGCGATTCGCTCCAACGAACTGGCTGACAGTGCATCCACTAAAGTTACCGAGGCTGCCCGTGTGGCAAACCAAGCTCAACAGCTTTCCAAATTCTTTGAGTAATCAAACAGGAGAAAccattatgatcgtttctgacatcgaagctaacgccctcttaga                                                                                                                                                                                                                                                              >  1:142177‑M1/1‑258 (MQ=255)`  
                                           `ttCTAATACGACTCACTAAAGGAGACACACCATGTTCAAACTGATTAAGAAGTTAGGCCAACTGCTGGTTCGTATGTACAACGAGGATGCCAAGCGACTGAACGATGAGGCTCGTCAAGAGGCCACAATGTCACGCGCTCTGGCGATTCGCTCCCACGAACTGGCTGACAGTGCATCCACTAAAGTTACCGAGTCTGCCCGTGTGGCAAACCAAGATCCACAGCTATCCAAATTCTTTGAGTAATCGAACACGAGAAAtcaatatgatcgtttcttacagctaagctaacgacctctttgc                                                                                                                                                                                                                                                              >  1:44010‑M1/1‑258 (MQ=255)`  
                                            `tCTAATACGACTCACTAAAGGAGACACACCATGTTCAAACTGATTAAGAAGTTAGGCCAACTGCTGGTTCGTATGTACAACGTGGAAGCCAAGCGACTGAACGATGAGGCTCGTAAAGAGGCCACACAGTCACGCGCTCTGGCGATTCGCTCCAACGAACTGGCTGACAGTGCATCCACTAAAGTTACCGAGGCTGCCCGTGTGGCAAACCAAGCTCAACAGCTTTCCAAATTCTTTGAGTAATCAAACAGGAGAAAtcaatatgatcgtttctgacatcgaagctaacgccctcttagag                                                                                                                                                                                                                                                             <  1:49591‑M1/301‑45 (MQ=255)`  
                                            `tCTAATACGACTCACGAAAGGAGAAACACCAAGTACAAACTGATTGAGAAATTAGGCCAACTGCTAGTTCGTAAGTACAACGTGGAAGCCAAGCGACTGAACGATGAGGCTCGTAAATAGGCCACACAATCGCGCTCTCTGGCAATTCGCTCCAACGAACAGGCTGACAGTGCATCCACTAAAGGTACCGAGGCTGGCCGTGTGACAAACCAAGCTCAACAGCTTTCCAAATTCTTTGAGTAATCAAACAGGAGAAAtcaatatgatcgtttctgacatcgaagctaacgccctcttagag                                                                                                                                                                                                                                                             <  1:215788‑M1/301‑45 (MQ=255)`  
                                            `agtaaTACGACTCACTAAAGGAGACAAACCATGTTCAAACTGATTAAGAAGTTAGGCCAACTGCTGGTTCGTATGTACAACGTGGAAGCCAAGCGACTGAACGATGAGGCTCGTAAAGAGGCCACACAGTCACGCGCTCTGGCGATTCGCTCCAACGAACTGGCTGACAGTGCATCCACTAAAGTTACCGAGGCTGCCCGTGTGGCAAACCAAGCTCAACAGCTTTCCAAATTCTTTGAGTAATCAAACAGGAGAAAccattatgatcgtttctgacatcgaagctaacgccctcttagag                                                                                                                                                                                                                                                             <  1:337920‑M1/299‑45 (MQ=255)`  
                                              `aaatACGACTCACTAAAGGAGACACACCATGTTCAAACTGATTAAGAAGTTAGGCCAACTTCTGGTTCGTATGTACAACGTGGAAGCCAAGCGACTGAACGATGAGGCTCGTAAAGAGGCCACACAGTCACGCGCTCTGGCGATTCGCTCCAACGAACTGGCTGACAGTGCATCCACTAAAGTTACCGAGGCTGCCCGTGTGGCAAACCAAGCTCAACAGCTTTCCAAATTCTTTGAGTAATCAAACAGGAGAAAccattatgatcgtttctgacatcgaagctaacgccctcttagagag                                                                                                                                                                                                                                                           <  1:345411‑M1/300‑47 (MQ=255)`  
                                               `aaTACGACTCACTAAAGGAGACACACCATGTTCAAACTGATTAAGAAGTTAGGCCAACTGCTGGTTCGTATGTACAACGTGGAAGCCAAGCGACTGAACGATGAGTCTCGTAAAGAGGCCACACAGTCACGCGCTCTGGCGATTCGCTCCAACGAACTGGCTGACAGTGCATCCACTAAAGTTACCGAGGCTGCCCGTGTGGCAAACCAAGCTCAACAGCTTTCCAAATTCTTTGAGTAATCAAACAGGAGAAAccattatgatcgtttctgacatcgaagctaacgccctcttagagctg                                                                                                                                                                                                                                                          >  1:136266‑M1/1‑254 (MQ=255)`  
                                               `aaTACGACTCACTAAAGGAGACACACCATGTTCAAACTGATTAAGAAGTTAGGCCAACTGCTGGTTCGTATGTACAACGTGGAAGCCAAGCGACTGAACGATGAGGCTCGTAAAGAGGCCACACAGTCACGCGCTCTGGCGATTCGCTCCAACGAACTGGCTGACAGTGCATCCACTAAAGTTACCGAGGCTGCCCGTGTGGCAAACCAAGCTCAACAGCTTTCCAAATTCTTTGAGTAATCAAACAGGAGAAAccattatgatcgtttctgacatcgaagctaacgccctcttagagagc                                                                                                                                                                                                                                                          >  1:208327‑M1/1‑254 (MQ=255)`  
                                                      `cTCACTAAAGGAGACACACCATGTTCAAACTGATTAAGAAGTTAGGCCAACTGCTGGTTCGTATGTACAACGTGGAAGCCAAGCGACTGAACGATGAGGCTCGTAAAGAGGCCACACAGTCACGCGCTCTGGCGATTCGCTCCAACGAACTGGCTGACAGTGCATCCACTAAAGTTACCGAGGCTGCCCGTGTGGCAAACCAAGCTCAACAGCTTTCCACATTCTTTGAGTAATCAAACAGGAGAAAtcaatatgatcgtttctgacatcgaagctaacgccctcttagagctgtctcttt                                                                                                                                                                                                                                                   >  1:325103‑M1/1‑247 (MQ=255)`  
                                                          `cTAAAGGAGACACACCATGTTCAAACTGATTAAGAAGTTAGGCCAACTGCTGGTTCGTATGTACAACGTGGAAGCCAAGCGACTGAACGATGAGGCTCGTAAAGAGGCCACACAGTCACGCGCTCTGGCGATTCGCTCCAACGAACTGGCTGACAGTGCATCCACTAAAGTTACCGAGGCTGCCCGTGTGGCAAACCAAGCTCAACAGCTTTCCAAATTCTTTGAGTAATCAAACAGGAGAAAtcaatatgatcgtttctgacatcaaagctaacgccctcttagagcgcttcactaagtt                                                                                                                                                                                                                                               >  1:212552‑M1/1‑243 (MQ=255)`  
                                                           `tAAAGGAGACACACCATGTTCAAACTGATTAAGAAGTTAGGCCAACTGCTGGTTCGTATGTACAACGTGGAAGCCAAGCGACTGAACGATGAGGCTCGTAAAGAGGCCACACAGTCACGCGCTCTGGCGATTCGCTCCAACGAACTGGCTGACAGTGCATCCACTAAAGTTACCGAGGCTGCCCGTGTGGCAAACCAAGCTCAACAGCTTTCCAAATTCTTTGAGTAATCAAACAGGAGAAAtcaatatgatcgtttctgacatcgaagctaacgccctcttagagagcgtcactaagttc                                                                                                                                                                                                                                              <  1:306580‑M1/301‑60 (MQ=255)`  
                                                            `aaaGGAGACACACCATGTTCAAACTGATTAAGAAGTTAGGCCAACTGCTGGTTCGTATGTACAACGTGGAAGCCAAGCGACTGAACGATGAGGCTCGTAAAGAGGCCACACAGTCACGCGCTCTGGCGATTCGCTCCCACGAACTGGCTGACAGTGCATCCACTAAAGTTACCGAGGCTGCCCGTGTGGCAAACCAAGCTCAACAGCTTTCCAAATTCTTTGAGTAATAAAACAGGAGAAAccattatgatcgcttctgaactcgaagctaacgctctcttagagagcgtcactaagcctg                                                                                                                                                                                                                                             >  1:148795‑M1/1‑241 (MQ=255)`  
                                                               `ggAGACACACCATGTTCAAACTGATTAAGAAGTTAGGCCAACTGCTAGTTCGTATGTACAACGTGGAAGCCAAGCGACTGAACGATGAGGCTCGTAAAGAGGCCACACAGTCACGCGCTCTGGCGATTCGCTCCAACGAACTGGCTGACAGTGCATCCACTAAAGTTACCGAGGCTGCCCGTGTGGCAAACCAAGCTCAACAGCTTTCCAAATTCTTTGAGTAATCAAACAGGAGAAAtcaatatgatcgtttctgacatcgaagctaacgccctcttagagagcgtcactaagttccacc                                                                                                                                                                                                                                          >  1:328425‑M1/1‑238 (MQ=255)`  
                                                                `gagaCACACCATGTTCAAACTGATTAAGAAGTTAGGCCAACTGCTGGTTCGTATGTACAACGTGGAAGCCAAGCGACTGAACGATGAGGCTCGTAAAGAGGCCACACAGTCACGCGCTCTGGCGATTCGCTCCAACGAACTGGCTGACAGTGCATCCACTAAAGTTACCGAGGCTGCCCGTGTGGCAAACCAAGCTCAACAGCTTTCCAAATTCTTTGAGTAATCAAACAGGAGAAAtcaatatgatcgtttctgacatcgaagctaacgccctcttagagagcgtcactaagttccactt                                                                                                                                                                                                                                         >  1:240323‑M1/1‑237 (MQ=255)`  
                                                                `gagaCACACCATGTTCAAACTGATTAAGAAGTTAGGCCAACTGCTGGTTCGTATGTACAACGTGGAAGCCAAGCGACTGAACGATGAGGCTCGTAAAGAGGCCACACAGTCACGCGCTCTGGCGATTCGCTCCAACGAACTGGCTGACAGTGCATCCACTAAAGTTACCGAGGCTGCCCGTGTGGCAAACCAAGCTCAACAGCTTTCCAAATTCTTTGAGTAATCAAACAGGAGAAAtcaatatgatcgtttctgacatcgaagctaacgccctcttagagagcgtcactaagttccactc                                                                                                                                                                                                                                         >  1:204982‑M1/1‑237 (MQ=255)`  
                                                                     `acacCATGTTCAAACTGATTAAGAAGTTAGGCCAACTGCTGGTTCGTATGTACAACGTGGAAGCCAAGCGACTGAACGATGAGGCTCGTAAAGAGGACACACAGTCACGCGCTCTGGCGATTCGCTCCAACGAACTGGCTGACAGTGCATCCACTAAAGTTACCGAGGCTGCCCGTGTGGCAAACCAAGCTCAACAGCTTTCCACATTCTTTGAGTAATCAAACAGGAGAAAtcaatctgatcgtttctgacatcgaagctacagccctcttagagagcttcactacgttccactgcgggg                                                                                                                                                                                                                                    >  1:56998‑M1/1‑232 (MQ=255)`  
                                                                      `cacCATGTTCAAACTGATTAAGAAGTTAGGCCAACTGCTGGTTCGTATGTACAACGTGGAAGCCAAGCGACTGAACGATGAGGCTCGTAAAGAGGCCACACAGTCACGCGCTCTGGCGATTCGCTCCAACGAACTGGCTGACAGTGCATCCACTAAAGTTACCGAGGCTGCCCGTGTGGCAAACCAAGCTCAACAGCTTTCCAAATTCTTTGAGTAATCAAACAGGAGAAAtcaatatgatcgtttctgacatcgaagctaacgccctcttagagagcgtcactaagttccactgcggggt                                                                                                                                                                                                                                   <  1:91619‑M1/301‑71 (MQ=255)`  
                                                                      `cacCATGTTCAAACTGATTAAGAAGTTAGGCCAACTGCTGGTTCGTATGTACAACGTGGAAGCCAAGCGACTGAACGATGAGGCTCGTAAAGAGGCCACACAGTCACGCGCTCTGGCGATTCGCTCCAACGAACTGGCTGACAGTGCATCCACTAAAGTTACCGAGGCTGCCCGTGTGGCAAACCAAGCTCAACAGCTTTCCAAATTCTTTGAGTAATCAAACAGGAGAAAtcaatatgatcgtttctgacatcgaagctaacgccctcttagagagcgtcactaagttccactgcggggt                                                                                                                                                                                                                                   >  1:162158‑M1/1‑231 (MQ=255)`  
                                                                      `aaccATGTTCAAACTGATTAAGAAGTTAGGCCAACTGCTGGTTCGTATGTACAACGTGGAAGCCAAGCGACTGAACGATGAGGCTCGTAAAGAGGCCACACAGTCACGCGCTCTGGCGATTCGCTCCAACGAACTGGCTGACAGTGCATCCACTAAAGTTGCCGAGGCTGCCCGTGTGGCAAACCAAGCTCAACAGCTTTCCAAATTCTTTGAGTAATCAAACAGGAGAAAtcaatatgatcgtttctgacatcgaagctaacgccctcttagagagcgtcactaagttccactgcggggt                                                                                                                                                                                                                                   <  1:62944‑M1/300‑71 (MQ=255)`  
                                                                        `ccATGTTCAAACTGATTAAGAAGTTAGGCCAACTGCTGGTTCGTATGTACAACGTGGAAGCCAAGCGACTGAACGATGAGGCTCGTAAAGAGGCCACACAGTCACGCGCTCTGGCGATTCGCTCCAACGAACTGGCTGACAGTGCATCCACTAAAGTTACCGAGGCTGCCCGTGTGGCAAACCAAGCTCAACAGCTTTCCAAATTCTTTGAGTAATCAAACAGGAGAAAtcaatatgatcgtttctgacatcgaagctaacgccctcttagagagcgtcactaagttccactgcggggtta                                                                                                                                                                                                                                 <  1:29165‑M1/301‑73 (MQ=255)`  
                                                                           `tGTTCAAACTGATTAAGAAGTTAGGCCAACTGCTGGTTCGTATGTACAACGTGGAAGCCAAGCGACTGAACGATGAGGCTCGTAAAGAGGCCACACAGTCACGCGCTCTGGCGATTCGCTCCAACGAACTGGCTGACAGTGCATCCACTAAAGTTACCGAGGCTGCCCGTGTGGCAAACCAAGCTCAACAGCTTTCCAAATTCTTTGAGTAATCAAACAGGAGAAAtcaatatgatcgtttctgacatcgaagctaacgccctcttagagagcgtcactaagttccactgcggggttatct                                                                                                                                                                                                                              >  1:26553‑M1/1‑226 (MQ=255)`  
                                                                           `tGTTCAAACTGATTAAGAAGTTAGGCCAACTGCTGGTTCGTATGTACAACGTGGAAGCCAAGCGACTGAACGATGAGGCTCGTAAAGAGGCCACACAGTCACGCGCTCTGGCGATTCGCTCCAACGAACTGGCTGACAGTGCATCCACTAAAGTTACCGAGGCTGCCCGTGTGGCAAACCAAGCTCAACAGCTTTCCAAATTCTTTGAGTAATCAAACAGGAGAAAccattatgatcgtttctgacatcgaagctaacgccctcttagagagcgtcactaagttccactgcggggttatct                                                                                                                                                                                                                              <  1:289726‑M1/301‑76 (MQ=255)`  
                                                                            `gTTCAAACTGATTAAGAAGTTAGGCCAACTGCTGGTTCGTATGTACAACGTGGAAGCCAAGCGACTGAACGATGAGGCTCGTAAAGAGGCCACACAGTCACGCGCTCTGGCGATTCGCTCCAACGAACTGGCTGACAGTGCATCCACTAAAGTTACCGAGGCTGCCCGTGTGGCAAACCAAGCTCAACAGCTTTCCAAATTCTTTGAGTAATCAAACAGGAGAAAtcattatgatcgtttctgacatcgaagctaacgccctcttagagagcgtcactaagttccactgcggggttatcta                                                                                                                                                                                                                             >  1:335316‑M1/1‑225 (MQ=255)`  
                                                                             `ttCAAACTGATTAAGAAGTTAGGCCAACTGCTGGTTCGTATGTACAACGTGGAAGCCAAGCGACTGAACGATGAGGCTCGTAAAGAGGCCACACAGTCACGCGCTCTGGTGATTCGCTCCAACGAACTGGCTGACAGTGCATCCACTAAAGTTACCGAGGCTGCCCGTGTGGCAAACCAAGCTCAACAGCTTTCCAAATTCTTTGAGTAATCAAACAGGAGAAAtcaatatgatcgtttctgacatcgaagctaacgccctcttagagagcgtcactaagttccactgcggggttatctac                                                                                                                                                                                                                            <  1:77609‑M1/301‑78 (MQ=255)`  
                                                                              `tCAAACTGATTAAGAAGTTAGGCCAACTGCTGGTTCGTATGTACAACGTGGAAGCCAAGCGACTGAACGATGAGGCTCGTAAAGAGGCCACACAGTCACGCGCTCTGGCGATTCGCTCCAACGAACTGGCTGACAGTGCATCCACTAAAGTTACCGAGGCTGCCCGTGTGGCAAACCAAGCTCAACAGCTTTCCAAATTCTTTGAGTAATCAAACAGGAGAAAtcaatatgatcgtttctgacatcgaagctaacgccctcttagagagcgtcactaagttccactgcggggttatctacg                                                                                                                                                                                                                           <  1:256612‑M1/301‑79 (MQ=255)`  
                                                                                `aaaCTGATTAAGAAGTTAGGCCAACTGCTGGTTCGTATGTACAACGTGGAAGCCAAGCGACTGAACGATGAGGCTCGTAAAGAGGCCACACAGTCGCGCGCTCTGGCGATTCGCTCCAACGAACTGGCTGACAGTGCATCCACTAAAGTTACCGAGGCTGCCCGTGTGGCAAACCAAGCTCAACAGCTTTCCAAATTCTTTGAGTAATCAAACAGGAGAAAtcaatatgatcgtttctgacatcgaagctaacgccctcttagagagcgtcactaagttccactgcggggttatctacgac                                                                                                                                                                                                                         <  1:152708‑M1/301‑81 (MQ=255)`  
                                                                                `aaaCTGATTAAGAAGTTAGGCCAACTGCTGGTTCGTATGTACAACGTGGAAGCCAAGCGACTGAACGATGAGGCTCGTAAAGAGGCCACACAGTCACGCGCTCTGGCGATTCGCTCCAACGAACTGGCTGACAGTGCATCCACTAAAGTTACCGAGGCTGCCCGTGTGGCAAACCAAGCTCAACAGCTTTCCAAATTCTTTGAGTAATCAAACAGGAGAAAtcaatatgatcgtttctgacatcgaagctaacgccctcttagagagcgtcactaagttccactgcggggttatctacgac                                                                                                                                                                                                                         <  1:179752‑M1/301‑81 (MQ=255)`  
                                                                                  `aCTGAGTAAGAAGTTAGGCCAACTGCTGGTTCGTATGTACAACGTGGAAGCCAAGCGACTGAACGATGAGGCTCGTAAAGAGGCCACACAGTCACGCGCTCTGGCGATTCGCTCCAACGAACTGGCTGACAGTGCATCCACTAAAGTTACCGAGGCTGCCCGTGTGGCAAACCAAGCTCAACAGCTTTCCAAATTCTTTGAGTAATCAAACAGGAGAAAtcaatatgatcgtttctgacatcgaagctaacgccctcttagagagcgtcactaagttccacttcggggttatctacgacta                                                                                                                                                                                                                       <  1:114016‑M1/301‑83 (MQ=255)`  
                                                                                     `tatTAAGAAGTCAGCCCAACTGCTGGTTCGTATGTACAACGTGGAAGCCAAGCGACTGAACGATGAGGCTCTTAAAGAGGCCACACAGTCACGCGCTCTGGCGATTCGCTCCAACGAACTGGCTGACAGTGCATCCACTAAAGTTACCGAGGCTGCCCGTGTGGCAAACCAAGCTCAACAGCTTTCCAAATTCTTTGAGTAATCAAACAGGAGAAAtcaatatgatcgtttctgacatcgaagctaacgccctcttagagagcgtcactaagttccactgcggggttatctacgactactc                                                                                                                                                                                                                    <  1:83534‑M1/300‑86 (MQ=255)`  
                                                                                     `gATTAAGAAGTTAGGCCAACTGCTGGTTCGTATGTACAACGTGGAAGCCAAGCGACTGAACGATGAGGCTCGTAAAGAGGCCACACAGTCACGCGCTCTGGCGATTCGCTCCAACGAACTGGCTGACAGTGCATCCACTAAAGTTACCGAGGCTGCCCGTGTGGCAAACCAAGCTCAACAGCTTTCCAAATTCTTTGAGTAATCAAACAGGAGAAAccattatgatcgtttctgacatcgaagctaacgccctcttagagagcgtcactaagttccactgcggggttatctacgactactc                                                                                                                                                                                                                    <  1:278863‑M1/301‑86 (MQ=255)`  
                                                                                      `aTTAAGAAGTTAGGCCAACTGCTGGTTCGTATGTACAACGTGGAAGCCAAGCGACTGAACGATGAGGCTCGTAAAGAGGCCACACAGTCACGCGCTCTGGCGATTCGCTCCAACGAACTGGCTGACAGTGCATCCACTAAAGTTACCGAGGCTGCCCGTGTGGCAAACCAAGCTCAACAGCTTTCCAAATTCTTTGAGTAATCAAACAGGAGAAAtcaatatgatcgtttctgacatcgaagctaacgccctcttagaaagcgtcactaagttccactgcggggttatctacgactacccc                                                                                                                                                                                                                   >  1:247561‑M1/1‑215 (MQ=255)`  
                                                                                        `tAAGAAGTTAGGCCAACGGCTGGTTCGTATGTACAACGTGGAAGCCAAGCGACTGAACGATGAGTCTCGTAAAGAGGCCACACAGTCACGCGCTCTGGCGATTCGCTCCAACGAACTGGCTGACAGTGCATCCACTAAAGTTACCGAGGCTGCCCGTGTGGCAAACCAAGCTCAACAGCTTTCCAAATTCTTTGAGTAATCAAACAGGAGAAAtcaatatgatcgtttctgacatcgaagctaacgccctcttagagagcgtcactaagttccactgcggggttatctacgactactccac                                                                                                                                                                                                                 <  1:194694‑M1/301‑89 (MQ=255)`  
                                                                                        `aaagagacagggccaactgctgGTTCGTATGTACAACGTGGAAGCCAAGCGACTGAACGATGAGGCTCGTAAAGAGGCCACACAGTCACGCGCTCTGGCGATTCGCTCCAACGAACTGGCTGACAGTGCATCCACTAAAGTTACCGAGGCTGCCCGTGTGGCAAACCAAGCTCAACAGCTTTCCAATTTCTTTGAGTAATCAAACAGGAGAAAtcaatatgatcgtttctgacatcgaagctaacgccctcttagagagcgtcactaagttccactgcggggttatctacgactactccac                                                                                                                                                                                                                 <  1:37738‑M1/291‑89 (MQ=255)`  
                                                                                        `aaagaagtTAGGCCAACTGCTGGTTCGTATGTACAACGTGGAAGCAAAGCGACTGAACGATGAGGCTCGTAAAGAGGCCACACAGTCACGCGCTCTGGCGATTCGCTCCAACTAACTGGCTGACAGTGCATCCACTAAAGTTACCGAGGCTTCCCGTGTGGCAAACCAAGCTCAACAGATTTCCAAATTCTTTGAGTAATCAAACAGGAGAAAtcaatatgatcgtttctgacatcgaagctaacgccctcttagagagcgtcactaagttccactgcggggttatctacgactactccac                                                                                                                                                                                                                 <  1:70660‑M1/300‑89 (MQ=255)`  
                                                                                           `gaagTTAGGCCAACTGCTGGTTCGTATGTACAACGTGGAAGCCAAGCGACTGAACGATGAGGCTCGTAAAGAGGCCACACAGTGACGCGCTCTGGCGATTCGCTCCAACGAACTGGCTGACAGTGCATCCACTAAAGTTACCGAGGCTGCCCGTGTGGCAAACCAAGCTCAACAGCTTTCCAAATTCTTTGAGTAATCAAACAGGAGAAAtcaatatgatcgtttctgacatcgaagctaacgccctcttagagagcgtcactaagttccactgcggggttatctacgactactccaccgc                                                                                                                                                                                                              <  1:269972‑M1/301‑92 (MQ=255)`  
                                                                                           `gaagTTAGGCCAACTGCTGGTTCGTATGTACAACGTGGAAGCCAAGCGACTGAACGATGAGGCTCGTAAAGAGGCCACACAGTCACGCGCTCTGGCGATTCGCTCCAACGAACTGGCTGACAGTGCATCCACTAAAGTTACCGAGGCTGCCCGTGTGGCAAACCAAGCTCAACAGCTTTCCAAATTCTTTGAGTAATCAAACAGGAGAAAtcaatatgatcttttctgacatcgaagctaacgccctcttagagagcgtcactaagttccactgcggggttatctacgacctgtctcttat                                                                                                                                                                                                              >  1:117947‑M1/1‑210 (MQ=255)`  
                                                                                           `gaagTTAGGCCAACTGCTGGTTCGTATGTACAACGTGGAAGCCAAGCGACTGAACGATGAGGCTCGTAAAGAGGCCACACAGTCACGCGCTCTGGCGATTCGCTCCAACGAACTGGCTGACAGTGCATCCACTAAAGTTACCGAGGCTGCACGTGTGGCAAACCAAGCTCAACAGCTTTCCAAATTCTTTGAGTAATCAAACAGGAGAAAtcaatatgatcgtttctgacatcgaagcaaacgccctcttagagagcgttactaagttccacggcggggttatatacgactactccaccgc                                                                                                                                                                                                              >  1:104287‑M1/1‑210 (MQ=255)`  
                                                                                           `aaagtTAGGCCAACTGCTGGTTCGTATGTACAACGTGGAAGCCAAGCGACTGAACGATGAGGCTCGTACAGAGGCCGCACAGTCACGCGCTCTGGCGATTCGCTCCAACGAACTGGCTGACAGTGCATCCACTAAAGTTACCGAGACTGCCCGTGTGGCAAACCAAGCTCAACAGCTTTCCAAATTCTTTGAGTAATCAAACAGGAGAAAtcaatatgatcgtttctgacctcgaagctaacgccctcttagagagcgtcactaagttccactgcggggttatctacgactactccaccgc                                                                                                                                                                                                              <  1:53516‑M1/300‑92 (MQ=255)`  
                                                                                             `agTTAGGCCAACTGCTGGTTCGTATGTACAACGTGGAAGCCAAGCGACTGAACGATGAGGCTCGTAAAGAGGCCACACAGTCACGCGCTCTGGCGATTCGCTCCAACGAACTGGCTGACAGTGCATCCACTAAAGTTACCGAGGCTGCCCGTGTGGCAAACCAAGCTCAACAGCTTTCCAAATTCTTTGAGTAATCAAACAGGAGAAAccattatgatcgtttctgacatcgaagctaacgccctcttagagagcgtcactaagttccactgcggggttatctacgcctactccaccgctg                                                                                                                                                                                                            >  1:164885‑M1/1‑208 (MQ=255)`  
                                                                                               `ttAGGCCAACTGCTGGTTCGTATGTACAACGTGGAAGCCAAGCGACTGAACGATGAGGCTCGTAAAGAGGCCACACAGTCACGCGCTCTGGCGATTCGCTCCAACGAACTGGCTGACAGTGCATCCACTAAAGTTACCGAGGCTGCCCGTGTGGCAAACCAAGCTCAACAGCTTTCCAAATTCTTTGAGTAATCAAACAGGAGAAAtcaatatgatcgtttctgacatcgaagctaacgccctcttagagagcgtcactaagttccactgcggggttatctacgactactccaccgctgag                                                                                                                                                                                                          <  1:74698‑M1/301‑96 (MQ=255)`  
                                                                                                `tAGGCCAAGTGCTGGTTCTTATGTACAACGTGGAATCCAAGCGACTGAACGATGAGGCGCGTAAAGAGGCCACAAAGTCACGCGCTCTGGCGATTCGCTCCAACGAACTGGCTGACAGTGCATCCACTAAAGTTACCGAGGCTGCCCGTGTGGCAAACCAAGCTCAACAGCTTTCCAAATTCTTTGAGTAATCAAACAGGAGAAAtcaatatgatcgtttctgacatcgaagctaacgccctcttagagagcgtcactaagttccactgcggggttatctacgactactccaccgctgagt                                                                                                                                                                                                         <  1:131284‑M1/301‑97 (MQ=255)`  
                                                                                                  `ggCCAACTGCTGGTTCGTATGTACAACGTGGAAGCCAAGCGACTGAACGATGAGGCTCGTAAAGAGGCCACACAGTCACGCGCTCTGGCGATTCGCTCCAACGAACTGGCTGACAGTGCATCCACTAAAGTTACCGAGGCTGCACGTGTGGCAAACCAAGCTCAACAGCGTTCCAAATTCTTTGAGTAATCAAACAGGAGAAAccattatgatcgtttctgacatcgaagcgaacgccctcttagagagcgtcattaagttccactgcggggttatctacgactactccaccgatgagtac                                                                                                                                                                                                       >  1:202416‑M1/1‑203 (MQ=255)`  
                                                                                                     `cAACTGTTGGTTCGATTGGACAACGTGGCAGCCAAGCGACTGAACGATGAGGCTCGTAAAGAGGCCACACAGTCACGCGCTCTGGCGATTCGCTCCAACGAACTGGCTGACAGTGCATCCACTAAAGTTACCGAGGCTGCCAGTGTGGCAAACCAAGCTCAACAGCTTTCCAAAATCTTTGAGTAATCAAACAGGAGAAAccattatgatcgtttctgacatcgaagctaacgccctcttagagagcgtcactaagttccactgcggggttatctacgactactccaccgctgagtacgta                                                                                                                                                                                                    <  1:123947‑M1/301‑102 (MQ=255)`  
                                                                                                     `cAACTGCTGGTTCGTATGTACAACGTGGAAGCCAAGCGACTGAACGATGAGGCTCGTAAAGAGGCCACACAGTCACGCGCTCTGGCGATTCGCTCCAACGAACTGGCTGACAGTGCATCCACTAAAGTTACCGAGGCTGCCCGTGTGGCAAACCAAGCTCAACAGCTTTCCAAATTCTTTGAGTAATCAAACAGGAGAAAtcaatatgatcgtttctgacatcgaagctaacgccctcttagagagcgtcactaagttccactgcggggttatctacgactactccaccgctgagtacgta                                                                                                                                                                                                    <  1:236008‑M1/301‑102 (MQ=255)`  
                                                                                                       `gtgtataagagacaggtacaacgtggaagccAAGCGACTGAACGATGAGGCTCGTAAAGAGGCCACACAGTCACGCGCTCTGGCGATTCGCTCCAACGAACTGGCTGACAGTGCATCCACTAAAGTTACCGAGGCTGCCCGTGTGGCAAACCAAGCTCAACAGCTTTCCAAATTCTTTGAGTAATCAAACAGGAGAAAtcaatatgatcgtttctgacatcgaagctaacgccctcttagagagcgtcactaagttccactgcggggttatctacgactactccaccgctgagtacgtaag                                                                                                                                                                                                  <  1:2725‑M1/286‑104 (MQ=255)`  
                                                                                                        `ctggtgGTTAGTATGTACAACGGGGAAGCCAAGCGACTGAACGATGAGGCTCGTAAAGAGGCCACACAGTCACGCGCTCTGGCGATTCGATCCAACGAACTGGCTGACAGTGCATCCACTAAAGTTACCGAGGCTGCCCGTGTGGCAAACCAAGCTCAACAGCTTTCCAAATTCTTTGAGAAATCAAACAGGAGAAAccattatgatcgtttctgacatcgaagctaacgccctcttagagagcgtcactaagttccactgcggggttatctacgactactccaccgctgagtacgtaagc                                                                                                                                                                                                 <  1:219472‑M1/301‑105 (MQ=255)`  
                                                                                                         `ctgttggtcggaagtaaaaaggggaaagcaagcgactgaacgatgaggctcgtaaagAGGCCACACAGTCACGCGCTCTGGCGATTCGCTCCAACGAACTGGCTGACAGTGCATCCACTAAAGTTACCGAGGCTGCCCGTGTGGCAAACCAAGCTCAACAGCTTTCCAAATTCTTTGAGTAATCAAACAGGAGAAAtcaatatgatcgtttctgacatcgaagctaacgccctcttagagagcgtcactaagttccactgcggggttatctacgactactccaccgctgagtacgtaagct                                                                                                                                                                                                <  1:110041‑M1/273‑106 (MQ=255)`  
                                                                                                                 `cGTATGTACAACGTGGAAGCCAAGCGACTGAACGATGAGGCTCGTAAAGAGGCCACACAGTCACGCGCTCTGGCGATTCGCTCCAACGAACTGGCTGACAGTGCATCCACTAAAGTTACCGAGGCTGCCCGTGTGGCAAACCAAGCTCAACAGCTTTCCAAATTCTTTGAGTAATCAAACAGGAGAAAtcaatatgatcgtttctgacatcgaagctaacgccctcttagagagcgtcactaagttccactgcggggttatctacgactactccaccgctgagtacgtaagctaccgtccg                                                                                                                                                                                        >  1:28742‑M1/1‑188 (MQ=255)`  
                                                                                                                 `cGTATGTACAACGTGGAAGCCAAGCGACTGAACGATGAGGCTCGTAAAGAGGCCACACAGTCACGCGCTCTGGCGATTCGCTCCAACGAACTGGCTGACAGTGCATCCACTAAAGTTACCGAGGCTGCCCGTGTGGCAAACCAAGCTCAACAGCTTTCCAAATTCTTTGAGTAATCAAACAGGAGAAAtcaatatgatcgtttctgacatcgaagctaacgccctcttagagagcgtcactaagttccactgcggggttatctacgactactccaccgctgagtacgtaagctaccgtccg                                                                                                                                                                                        >  1:1708‑M1/1‑188 (MQ=255)`  
                                                                                                                    `aTGTACAACGTGGAAGCCAAGCGACTGAACGATGAGGCTCGTAAAGAGGCCACACAGTCACGCGCTCTGGCGATTCGCTCCAACGAACTGGCTGACAGTGCATCCACTAAAGTTACCGAGGCTGCCCGTGTGGCAAACCAAGCTCAACAGCTTTCCAAATTCTTTGAGTAATCAAACAGGAGAAAtcaatatgatcgtttctgacatcgaagctaacgccctcttagagagcgtcactaagttccactgcggggttatctacgactactccaccgctgagtacgtaagctaccgtccgagt                                                                                                                                                                                     <  1:59326‑M1/301‑117 (MQ=255)`  
                                                                                                                      `gTACAACGTGGAAGCCAAGCGACTGAACGATGAGGCTCGTAAAGAGGCCACACAGTCACGCGCTCTGGCGATTCGCTCCAACGAACTGGCTGACAGTGCATCCACTAAAGTTACCGAGGCTGCCCGTGTGGCAAACCAAGCTCAACAGCTTTCCAAATTCTTTGAGTAATCAAACAGGAGAAAccattatgatcgtttctgacatcgaagctaacgccctcttagagagcgtcactaagttccactgcggggttatctacgactactccaccgctgagtacgtaagctaccgtccgagtga                                                                                                                                                                                   >  1:107419‑M1/1‑183 (MQ=255)`  
                                                                                                                      `gTACAACGTGGAAGCCAAGCGACTGAACGATGAGGCTCGTAAAGAGGCCACACAGGCACGCGCTCTGGCGATTCGCTCCAACGAACTGGCTGACAGTGCATCCACTAAAGTTACCGAGGCTGCCCGTGTGGCAAACCAAGCTCAACAGCTTTCAAAATTCTTTGAGTAATCAAACAGGAGAAAccattatgatcgtttctgacatcgaagctaacgccctcttagagagcgtcactaagttcccctgcggggttatctacgactactccacagctgagtacgtaagctaccgtccgagtgg                                                                                                                                                                                   >  1:303891‑M1/1‑183 (MQ=255)`  
                                                                                                                           `aCGTGGAAGCCAAGCGACTGAACGATGAGGCTCGTAAAGAGGCCACACAGTCACGCGCTCTGGCGATTCGCTCCAACGAACTGGCTGACAGTGCATCCACTAAAGTTACCGAGGCTGCCCGTGTGGCAAACCAAGCTCAACAGCTTTCCAAATTCTTTGAGTAATCAAACAGGAGAAAtcaatatgatcgtttctgacatcgaagctaacgccctcttagagagcgtcactaagttccactgcggggttatctacgactactccaccgctgagtacgtaagctaccgtccgagtgacttcg                                                                                                                                                                              >  1:226123‑M1/1‑178 (MQ=255)`  
                                                                                                                            `cGTGGAAGCCAAGCGACTGAACGATGAGGCTCGTAAAGAGGCCACACAGTCACGCGCTCTGGCGATTCGCTCCAACGAACTGGCTGACAGTGCATCCACTAAAGTTACCGAGGCTGCCCGTGTGGCAAACCAAGCTCAACAGCTTTCCAAATTCTTTGAGTAATCAAACAGGAGAAAtcaatatgatcgtttctgacatcgaagctaacgccctcttagagagcgtcactaagttccactgcggggttatctacgactactccaccgctgagtacgtaagctaccgtccgagtgacttcgg                                                                                                                                                                             >  1:199862‑M1/1‑177 (MQ=255)`  
                                                                                                                            `cGTGGAAGCCAAGCGACTGAACGATGAGGCTCGTAAAGAGGCCACACAGTCACGCGCTCTGGCGATTCGCTCCAACGAACTGGCTGACAGTGCATCCACTAAAGTTACCGAGGCTGCCCGTGTGGCAAACCAAGCTCAACAGCTTTCCAAATTCTTTGAGTAATCAAACAGGAGAAAccattatgatcgtttctgacatcgaagctaacgccctcttagagagcgtcactaagttccactgcagggttatctacgactactccaccgctgagtacgtaagctaccgtccgagtgacttcgt                                                                                                                                                                             >  1:253602‑M1/1‑177 (MQ=255)`  
                                                                                                                                                     `gAGGCTCGTAAAGAGGCCACACAGTCACGCGCTCTGGCGATTCGCTCCAACGAACTGGCTGACAGTGCATCCACTAAAGTTACCGAGGCTGCCCGTGTGGCAAACCAAGCTCAACAGCTTTCCAAATTCTTTGAGTAATCAAACAGGAGAAAtcaatatgatcgtttctgacatcgaagctaacgccctcttagagagcgtcactaagttccactgcggggttatctacgaatactccaccgctgagtacgtaagctaccgtccgattgacttcgttgcgtatctggatgcgctggaagcc                                                                                                                                                    >  1:156208‑M1/1‑152 (MQ=255)`  
                                                                                                                                                           `cGTAAAGAGGCCACACAGTCACGCGCTCTGGCGATTCGCTCCAACGAACTGGCTGACAGTGCATCCACTAAAGTTACCGAGGCTGCCCGTGTGGCAAACCAAGCTCAACAGCTTTCCAAATTCTTTGAGTAATCAAACAGGAGAAAtcaatatgatcgtttctgacatctaagctaacgccctcttagagagcgtcactaagttccactgcggggttatctacgactactccaccgctgagtacgtaagctaccgtccgactgacttcggtgcgtatctggactgtctcttatacacatct                                                                                                                                              >  1:230043‑M1/1‑146 (MQ=255)`  
                                                                                                                                                                `agagGCCACACAGTCACGCGCTCTGTCGATTCGCTCCAACGAACTGGCTGACAGTGCATCCACTAAAGTTACCGAGGCTGCCCGTGTGGCAAACCAAGCTCAACAGCTTTCCAAATTCTTTGAGTAATCAAACAGGAGAAAtcaatatgatcgtttctgacatcgaagctaacgccctcttagagagcgtcactaagttccactgcggggttatctacgactactccaccgctgagtacgtaagctaccgtccgagtgacttcggtgcgtatctggatgcgctggaagccgaggttgcacg                                                                                                                                         <  1:133969‑M1/301‑161 (MQ=255)`  
                                                                                                                                                                 `gagGCCACACAGTCACGCGCTCTGGCGATTCGCTCCAACGAACTGGCTGACAGTGCATCCACTAAAGTTACCGAGGCTGCCCGTGTGGCAAACCAAGCTCAACAGCTTTCCAAATTCTTTGAGTAATCAAACAGGAGAAAtcaatatgatcgtttctgacatcgaagctaacgccctcttagagagcgtcactaagttccactgcggggttatctacgactactccaccgctgagtacgtaagctaccgtccgagtgacttcggtgcgtatctggatgcgctggaagccgaggttgcacga                                                                                                                                        <  1:321395‑M1/301‑162 (MQ=255)`  
                                                                                                                                                                 `gagGCCACACAGTCACGCGCTCTGGCGATTCGCTCCAACGAACTGGCTGACAGTGCATCCACTAAAGTTACCGAGGCTGCCCGTGTGGCAAACCAAGCTCAACAGCTTTCCAAATTCTTTGAGTAATCAAACAGGAGAAAtcaatatgatcgtttctgacatcgaagctaacgccctcttagagagcgtcactaagttccactgcggggttatctacgactactccaccgctgagaacgtaagctaccgtccgagtgacttcggtgcgtatctggatgcgctggaagccgaggttgcacga                                                                                                                                        >  1:28421‑M1/1‑140 (MQ=255)`  
                                                                                                                                                                   `ggCCACACAGTAACGGGGTCTGGCGATTCGCTCCAACGAACTGGCTGACAGTGCATCCACTAAAGTTACCGAGGCTGCCCGTGTGGCAAACCAAGCTCAACAGCTTTCCAAATTCGTTGAGTAATCAAACAGGAGAAAtcaatatgatcgtttctgacatcggagctaacgccctcttagagagcgtcactaagtgccactgcggggttatctacgactactccaccgctgagtacgtaagctaccgtccgagtgacttcggtgcgtatctggatgcgctggaagccgaggttgcacgagg                                                                                                                                      <  1:31859‑M1/301‑164 (MQ=255)`  
                                                                                                                                                                           `aGTCACGCGCTCTGGCGATTCGCTCCAACGAACTGGCTGACAGTGCATCCACTAAAGTTACCGAGGCTGCCCGTGTGGCAAACCAAGCTCAACAGCTTTCCAAATTCTTTGAGTAATCAAACAGGAGAAAccattatgatcgtttctgacatcgaagctaacgccctcttagagagcgtcactaagttccactgcggggttatctacgactactccaccgctgagtacgtaagctaccgtccgagtgacttcggtgcgtatctggatgcgctggaagccgaggttgcacgaggcggtcttt                                                                                                                              >  1:312327‑M1/1‑130 (MQ=255)`  
                                                                                                                                                                            `gTCACGCGCTCTGGCGATTCGCTCCAACGAACTGGCTGACAGTGCATCCACTAAAGTTACCGAGGCTGCCCGTGTGGCAAACCAAGCTCAACAGCTTTCCAAATTCTTTGAGTAATCAAACAGGAGAAAtcaatatgatcgtttctgacatcgaagctaacgccctcttagagagcgtcactaagttccactgcggggttatctacgactactccaccgctgagtacgtaagctaccgtccgagtgacttcggtgcgtatctggattcgctggaagccgaggttgcacgaggcggtcttct                                                                                                                             >  1:187729‑M1/1‑129 (MQ=255)`  
                                                                                                                                                                            `gTCACGCGCTCTGGCGATTCGCTCCAACGAACTGGCTGACAGTGCATCCACTAAAGTTACCGAGGCTGCCCGTGTGGCAAACCAAGCTCAACAGCTTTCCAAATTCTTTGAGTAATCAAACAGGAGAAAtcaatatgatcgtttctgacatcgaagctaacgccctcttagagagcgtcactaagttccactgcggggttatctacgactactccaccgctgagtacgtaagctaccgtccgagtgacttcggtgcgtatctggatgcgctggaagccgaggttgcacgaggcggtcttat                                                                                                                             >  1:297397‑M1/1‑129 (MQ=255)`  
                                                                                                                                                                            `gTCACGCGCTCTGGCGATTCGCTCCAACGAACTGGCTGACAGTGCATCCACTAAAGTTACCGAGGCTGCCCGTGTGGCAAACCAAGCTCAACAGCTTTCCAAATTCTTTGAGTAATCAAACAGGAGAAAtcaatatgatcgtttctgacatcgaagctaacgccctcttagagagcgtcactaagttccactgcggggttatctacgactactccaccgctgagtacgtaagctaccgtccgagtgacttcggtgcgtatctggatgcgctggaagccgaggttgcacgaggcggtcttat                                                                                                                             >  1:2757‑M1/1‑129 (MQ=255)`  
                                                                                                                                                                              `cACGCGCTCTGGCGATTCGCTCCAACGAACTGGCTGACAGTGCATCCACTAAAGTTACCGAGGCTGCCCGTGTGGCAAACCAAGCTCAACAGCTTTCCAAATTCTTTGAGTAATCAAACAGGAGAAAtcaatatgatcgtttctgacatcgaagctaacgccctcttagagagcgtcactaagttccactgcggggttatctacgactactccaccgctgagtacgtaagctaccgtccgagtgacttcggtgcgtatctggatgcgctggaagccgaggttgcacgaggcggtcttattg                                                                                                                           <  1:86934‑M1/301‑175 (MQ=255)`  
                                                                                                                                                                              `cACGCGATCTGGTGATTCGCTCCAACGAACTGGCTGACTGTGCATCCACTAAAGTTACCGATGCACCCCGTGTGGCAAACCAAGCTCAACAGCTTTCCAAATTCTGTGAGTAATCAAACAGGAGAAAccattatgatcgtttctgacatcgaagctaacgccccctgagagagcgtcactaagttccactgcggggttatctacgactactccaccgctgagtacggaagctaccgtccgagtgacttcggtgcgtatctggatgcgctggaagccgaggttgcacgaggcggtcttattg                                                                                                                           <  1:145856‑M1/301‑175 (MQ=255)`  
                                                                                                                                                                                `cgcgCTCTGGCGATTCGCTCCAACGAACTGGCTGACAGTGCATCCACTAAAGTTACCGAGGCTGCCCGTGTGGCAAACCAAGCTCAACAGCTTTCCAAATTCTTTGAGTAATCAAACAGGAGAAAtcaatatgatcgtttctgacatcgaagctaacgccctcttagagagcgtcactaagttccactgcggggttatctacgactactccaccgctgagtacgtaagctaccgtccgagtgacttcggtgcgtatctggatgcgctggaagccgaggttgcacgaggcgttcttattgtg                                                                                                                         >  1:182982‑M1/1‑125 (MQ=255)`  
                                                                                                                                                                                                       `cGAACTGGCTGACAGTGCATCCACTAAAGTTACCGAGGCTGCCCGTGTGGCAAACCAAGCTCAACAGCTTTCCAAATTCTTTGAGTAATCAAACAGGAGAAAtcaatatgatcgtttctgacatcgaagctaacgccctcttagagagcgtcactaagttccactgcggggttatctacgactactccaccgctgagtacgtaagctaccgtccgagtgacttcggtgcgtatctggatgcgctggaagccgaggttgcacgaggcggtcttattgtgttccacaacggtcacaagtatga                                                                                                  <  1:281192‑M1/301‑200 (MQ=255)`  
                                                                                                                                                                                                         `aaCTGGCTGACAGTGCATCCACTAAAGTTACCGAGGCTGCCCGTGTGGCAAACCAAGCTCAACAGCTTTCCAAATTCTTTGAGTAATCAAACAGGAGAAAtcaatatgatcgtttctgacatcgaagctaacgccctcttagagagcgtcactaagttccactgcggggttatctacgactactccaccgctgagtacgtaagctaccgtccgagtgacttcggtgcgtatctggatgcgctggaagccgaggttgcacgaggcggtcttattgtgttccacaacggtcacaagtatgacg                                                                                                >  1:260625‑M1/1‑100 (MQ=255)`  
                                                                                                                                                                                                         `aaCTGGCTGACAGAGCATCCACGAAAGGTACCTAGGCCCCCCGTGAGGCAAACCAAGCTCAACAGCTTTCCAAATGCGTTGAGGAAGCAAACAGGAGACAgcaatatgatcggttctgacatagaagctaacgccctcgtagagagcgtcactaagtcccactgcggggttatctacgactactccaccgctgcgtacgtaagctactgtccgagtgacttcggtgcgtatctggatgcgcttgaagccgaggttgcacgaggcggtcttattgtgttccacaacggtcacaagtatgacg                                                                                                <  1:97437‑M1/301‑202 (MQ=255)`  
                                                                                                                                                                                                             `ggCTGACAGTGCATCCACTAAAGTTACCGAGGCTGCCCGTGTGGCAAACCAAGCTCAACAGCTTTCCAAATTCTTTGAGTAATCAAACAGGAGAAAtcaatatgatcgtttctgacatcgaagctaacgccctcttagagagcgtcactaagttccactgcggggttatctacgactactccaccgctgagtacgtaagctaccgtccgagtgacttcggtgcgtatctggatgcgctggaagccgaggttgcacgaggcggtcttattgtgttccacaacggtcacaagtatgacgttcc                                                                                            >  1:223197‑M1/1‑96 (MQ=255)`  
                                                                                                                                                                                                                  `aCAGTGCATCCACTAAAGTTACCGAGGCTGCCCGTGTGGCAAACCAAGCTCAACAGCTTTCCAAATTCTTTGAGTAATCAAACAGGAGAAAtcaatatgatcgtttctgacatcgaagctaacgccctcttagagagcgtcactaagttccactgcggggttatctacgactactccaccgctgagtacgtaagctaccgtccgagtgacttcggtgcgtatctggatgcgctggaagccgaggttgcacgaggcggtcttattgtgttccacaacggtcacaagtatgacgttcctgcat                                                                                       <  1:245360‑M1/301‑211 (MQ=255)`  
                                                                                                                                                                                                                     `gTGCATCCACTAAAGTTACCGAGGCTGCCCGTGTGGCAAACCAAGCTCAACAGCTTTCCAAATTCTTTGAGTAATCAAACAGGAGAAAtcaatatgatcgtttctgacatcgaagctaacgccctcttagagagcgtcactaagttccactgcggggttatctacgactactccaccgctgagtacgtaagctaccgtccgagtgacttcggtgcgtatctggatgcgctggaagccgaggttgcacgaggcggtcttattgtgttccacaacggtcacaagtatgacgttcctgcattga                                                                                    >  1:250569‑M1/1‑88 (MQ=255)`  
                                                                                                                                                                                                                                 `aaGTTACCGAGGCTGCCCGTGTGGCAAATCAAGCTCAACAGCTTTCCAAATTCTTTGAGTAATCAAACAGGAGAAAccattatgatcgtttctgacatcgaagctaacgccctcttagagagcgtcactaagttccactgcggggttatctacgactactccaccgctgagtacgtaagctaccgtccgagtgacttcggttcgtatctggatgcgctggaagccgaggttgcacgaggcggtcttattgtgttccacaacggtcacaagtatgacgttcctgcattgaccaaactggcaa                                                                        >  1:89724‑M1/1‑76 (MQ=255)`  
                                                                                                                                                                                                                                 `aaGTTACCGAGGCTGCCCGTGTGGCAAACCAACCTCAACAGCTTTCCAAATTCTTTTAGTAATCAAACAGGAGAAAccattatgatcgtttctgacatcgaagctaacgccctcttagagagcgtcactaagttccactgcggggttatctacgactactccaccgcttattacgtaagctaccggccgagtgacttcggtgcgtatctggatgcgcttgaagccgaggttgcacgaggcggtcttattgtgttccacaacggtcacaagtatgacgttcctgcattgaccaaactggcaa                                                                        <  1:120676‑M1/301‑226 (MQ=255)`  
                                                                                                                                                                                                                                    `ttACCGAGGCTGCCCGTGTGGCAAACCAAGCTCAACAGCTTTCCAAAGTCTTTGAGTAATCAAACAGGAGAAAccattatgatcgtttctgacatcgaagctaacgacctcttagagagcgtcactaagttccactgcgtggttatctacgactactccaccgctgagtacgtaagctaccgtccgagtgacttcggtgcgtatctggatgcgctggaagccgaggttgcacgaggcggtcttattgtgttccacaacggtcacaagtatgacgttcctgcattgaccaaactggcaaagt                                                                     <  1:158999‑M1/301‑229 (MQ=255)`  
                                                                                                                                                                                                                                           `ggCTGCCAGTGTGGCAAACCAATCTAAACAGCTTTCTAAATGTTTGGAGTAATCAAATAGGTGAAGacaatattatcgtttctgacatcgaagctaacggcctcttagagatcgtcactaagttccgctgcgtggttatctactactactccaccgctgaggacgttagctaccgtccgagtgacttcggtgcgtatctggatgcgctggaagccaaggttgcacgaggcggtcttattgtgttccacaacggtcacaagtatgacgttcctgcattgaccaaactggcaaagttgcaatt                                                              <  1:232530‑M1/301‑236 (MQ=255)`  
                                                                                                                                                                                                                                                                `aGCGCAACATCTTTCCAAATTCTTTGGGTAATCAAACAGGAGAAAtcattatgatcgtttctgacatcgaagctaacgccctcttagagagcgtcactaagttccactgcggtgttatctacgactactccaccgctgagtacgtaagctaccgtccgagtgacttcggtgcgtatctggatgcgctggaagccgaggttgcacgaggcggtcttattgtgttccacaacggtcacaagtatgacgttcctgcattgaccaaactggcaaagttgcaattgaaccgagagttccaccttcc                                         <  1:46814‑M1/301‑257 (MQ=255)`  
                                                                                                                                                                                                                                                                         `gCTTTCCACATTCTTTGAGTAATCAAACAGGAGAAAtcaatatgatcgtttctgacatcgaagctaacgccctcttagagagcgtcactaagttccactgcggggttatctacgactactccaccgctgagtacgtaagctaccgtccgagtgcattctgtgcgtatctggattcgctggaagccgaggctgcacgaggaggtcttattgtgttccacaaccgtcacaagtataacgttcctgcattgaccaaactggcaaagttgcaatttaaccgagagtaacctgcctcctatacaca                                >  1:315695‑M1/1‑36 (MQ=255)`  
                                                                                                                                                                                                                                                                          `ctcgtgggctcggagatgtgtataagagacagaaaccattatgatcgtttctgacatcgaagctaacgccctcttagagagcgtcactaagttccactgcggggttatctacgactactccaccgctgagtacgtaagctaccgtccgagtgacttcggtgcgtatctggatgcgctggaagccgaggttgcacgaggcggtcttattgtgttccacaacggtcacaagtatgacgttcctgcattgaccaaactggcaaagttgcaattgaaccgagagttccaccttcctcgtgagaac                               <  1:115549‑M1/271‑267 (MQ=255)`  
                                                                                                                                                                                                                                                                             `tCCAAATTCTTTGAGTAATCAAACATGAGAAAtcaatatgatcgtttctgacatcgaagctaacgccctcttagagagcgtcactaagttccactgctgggttatctacgaatactccaccgctgagtacgtaagctaccgtccgagtgacttcggtgcgtatctggatgcgctggaagccgaggttgcacgaggcggtcttattgtgttccacaacggtcacaagtatgacgttcctgcattgaccaaactggcaaagttgcaattgaaccgagagttacaccttcctcgtgagaactgt                            >  1:128556‑M1/1‑32 (MQ=255)`  
                                                                                                                                                                                                                                                                                 `aaTTCATTGAGTAATCAAACAGGCGACAccattatgatcgtttctgacatcgaagctatcgtcctcttagagggcttcactaagttccactgcggagctatctacgactacgccaccgctgtgtacgtaagctaccgtccgagtgacttcggtgcgtatctggatgcgctggaagccgaggttgcacgaggcggtcttattgtgttccacaacggtcacaagtatgacgttcctgcattgaccaaactggcaaagttgcaattgaaccgagagttccaccttcctcgtgagaactgtattg                        <  1:93560‑M1/301‑274 (MQ=255)`  
                                                                                                                                                                                                                                                                                   `ttCTTTGAGTAATCAAACAGGAGAAAtcaatatgatcgtttctgacatcgaagctaacgccctcttagagagcgtcactaagttccactgcggggttatctacgactactccaccgctgagtacgtaagctaccgtccgagtgacttcggtgcgtatctggatgcgctggaagccgaggttgcacgaggcggtcttattgtgttccacaacggtcacaagtatgacgttcctgcattgaccaaactggcaaagttgcaattgaaccgagagttccaccttcctcgtgagaactgtattgaa                      >  1:294273‑M1/1‑26 (MQ=255)`  
                                                                                                                                                                                                                                                                                               `tCAAACAGGAGAAAccattatgatcgtttctgacatcgaagctaacgccctcttagaaagcgtcactaagttccactgcggggttatctacgactactccaccgctgagtacgtaagctaccgtccgagtgacttcggtgcgtatctggatgcgctggaagccgaggttgcacgaggcggtcttattgtgttccacaacggtcacaagtatgacgttcctgcattgaccaaactggcaaagttgcaattgaaccgagagttccaccttcctcgtgagaactgtatttacactgtatctttt          >  1:343458‑M1/1‑14 (MQ=255)`  
                                                                                                                                                                                                                                                                                               `attagagacagaaaccattatgatcgtttctgacatcgaagctaacgccctcttagagagcgtcactaagttccactgcggggttatctacgactactccaccgctgagtacgtaagctaccgtccgattgacttcggtgcgtatctggatgcgctgtaagccgaggttgcacgaggcggtcttattgtgttccacaacggtcacaagtatgacgttcctgcattgaccaaactggcaaagttgcaattgaaccgagagttccaccttcctcgtgagaactgtattgtcacccttgtgttg          <  1:300537‑M1/292‑288 (MQ=255)`  
                                                                                                                                                                                                                                                                                                  `aaCAGGAGAAAtcaatatgatcgtttctgacatcgaagctaacgccctcttagagagcgtcactaagttccactgcggggttatctacgactactccaccgctgagtacgtaagctaccgtccgagtgacttcggtgcgtatttggatgcgctggaagccgaggttgcacgaggcggtcttattgtgttccacaacggtcacaagtatgacgttcctgcattgaccaaactggcaaagttgcaattgaaccgagagttccaccttcctcgtgagaactgtattgacacccttgtgttgtca       <  1:33125‑M1/301‑291 (MQ=255)`  
                                                                                                                                                                                                                                                                                                      `ggAGAAAtcaatatgatcgtttctgacatcgaagctaacgccctcttagagagcgtcactaagttccactgctgggttatctacgactactccaccgctgagtacgtaagctaccgtccgagtgacttcggtgcgtatctggatgcgcttgaagccgcggttgcacgaggcggtcttattgtgttccacaacggtcacaagtatgacgttcctgcattgaccaaactggcaaagttgcaattgaaccgagagttccaccttcctcgtgagaactgtattgacccccttgtgttgtcacgtt   >  1:340911‑M1/1‑7 (MQ=255)`  
                                                                                                                                                                                                                                                                                                       `gagaAAccattatgatcgtttctgacatcgaagctaacgccctcttagagagcgtcactaagttccactgcggggttatctacgactactccaccgctgagtacgtaagctaccgtccgagtgacttcggtgcgtatctggatgcgctggaagccgaggttgcacgaggcggtcttattgtgttccacaacggtcacaagtatgacgttcctgcattgaccaaactggcaaagttgcaattgaaccgagagttccaccttcctcgtgagaactgtattgacacccttgtgttgtcacgttt  >  1:40012‑M1/1‑6 (MQ=255)`  

                                                                                                                                                                                                                                                                                                            `|`                                                                                                                                                                                                                                                                                                         
`TGACTTTCCAGACGACTACGAGAAGTTTCGCTGGAGAGTCCCATTCTAATACGACTCACTAAAGGAGACACACCATGTTCAAACTGATTAAGAAGTTAGGCCAACTGCTGGTTCGTATGTACAACGTGGAAGCCAAGCGACTGAACGATGAGGCTCGTAAAGAGGCCACACAGTCACGCGCTCTGGCGATTCGCTCCAACGAACTGGCTGACAGTGCATCCACTAAAGTTACCGAGGCTGCCCGTGTGGCAAACCAAGCTCAACAGCTTTCCAAATTCTTTGAGTAATCAAACAGGAGAAACCATTATGTCTAACGTAGCTGAAACTATCCGTCTATCCGATACAGCTGACCAGTGGAACCGTCGAGTCCACATCAACGTTCGCAACGGTAAGGCGACTATGGTTTACCGCTGGAAGGACTCTAAGTCCTCTAAGAATCACACTCAGCGTATGACGTTGACAGATGAGCAAGCACTGCGTCTGGTCAATGCGCTTACCAAAGCTGCCGTGACAGCAATTCATGAAGCTGGTCGCGTCAATGAAGCTATGGCTATCCTCGACAAGATTGATAACTAAGAGTGGTATCCTCAAGGTCG  >  NC_001604.1/13278‑13873`

  

Alignment Legend

Aligned base mismatch/match (shaded by quality score): `ATCG/ATCG` `< 3 ≤` `ATCG/ATCG` `< 10 ≤` `ATCG/ATCG` `< 23 ≤` `ATCG/ATCG` `< 34 ≤` `ATCG/ATCG` `< 38 ≤` `ATCG/ATCG`

Unaligned base: `atcg`    Masked matching base: `atcg`    Alignment gap: `‑`    Deleted base: `‑`
